# Comment on: A review of the use of time‐varying covariates in the Fine‐Gray subdistribution hazard competing risk regression model by Peter C. Austin, Aurélien Latouche, and Jason P. Fine

**DOI:** 10.1002/sim.8558

**Published:** 2020-08-04

**Authors:** Shunsuke Mori

**Affiliations:** ^1^ Department of Rheumatology, Clinical Research Center for Rheumatic Diseases National Hospital Organization Kumamoto Saishun Medical Center Kumamoto Japan

**Keywords:** clinical disease activity index, competing risks, drug retention, Fine‐Gray model, rheumatoid arthritis, time‐varying covariates

Sir, I read the article by Austin et al regarding the use of time‐varying covariates in the Fine‐Gray subdistribution hazard competing risk regression model.^1^ In that article, the authors indicated that the inclusion of internal time‐varying covariates in a Fine‐Gray model results in the loss of the ability to estimate the cumulative incidence function (CIF) or the effect of covariates on CIF. I agree with this conclusion. In addition, Austin et al presented several articles reporting time‐varying covariates in this model and concluded that these papers had inappropriately described time‐varying covariates as being associated with risk of the outcome. A paper that I recently published in *Rheumatology*, titled “Retention of tocilizumab with and without methotrexate during maintenance therapy for rheumatoid arthritis: the ACTRA‐RI cohort study,”^2^ was included in the authors' list. The authors pointed out that clinical disease activity index (CDAI) was incorporated into Fine‐Gray models although it was a time‐varying covariate. However, I believe that the authors have misunderstood my study design and data interpretation.

The efficacy of tocilizumab (TCZ), the first humanized monoclonal anti‐interleukin‐6 receptor antibody, has been demonstrated against moderate to severe rheumatoid arthritis (RA).^3^ The durability of TCZ monotherapy related to that of combination therapy with methotrexate (MTX) remains an important issue, however, given that, in daily practice, rheumatologists often encounter patients who are intolerant of MTX. To address this issue, I evaluated TCZ retention during maintenance therapy among RA patients who had achieved an initial improvement during the first year of treatment (induction therapy). For this purpose, I used an ongoing real‐world registry containing all RA patients who had begun TCZ treatment with or without MTX since April 2008. A total of 510 patients with high or moderate clinical disease activity index (CDAI >10) had started TCZ treatment through November 2016. Among these, 328 had achieved and maintained a CDAI50 response, defined as 50% improvement of CDAI, at the end of induction therapy. I referred to these patients as CDAI50 responders and followed them during maintenance therapy. Follow‐up of CDAI50 responders started on the first day of maintenance therapy and ended with TCZ discontinuation, loss to follow‐up, death, or the last follow‐up visit, whichever was first. The design of my study is shown in Figure 1.

**FIGURE 1:**
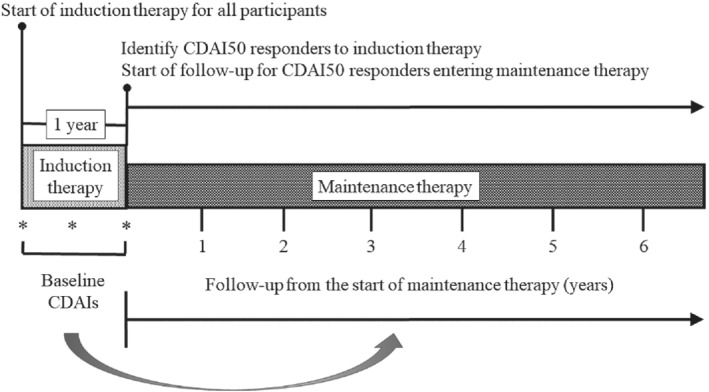
Study design. I first identified RA patients who had achieved a CDAI50 response, defined as a 50% improvement in CDAI, at the end of first‐year induction therapy (CDAI50 responders); I then followed these patients during maintenance therapy. As potential confounders, I used CDAI values measured at the start of induction therapy (an indicator of initial disease activity) as well as CDAI values measured at 6 and 12 months into this therapy (indicators of residual disease activity at or before the start of maintenance therapy). These were used as fixed baseline confounders for treatment effect (TCZ monotherapy vs combination therapy with MTX) on discontinuation during maintenance therapy. CDAI, clinical disease activity index; RA, rheumatoid arthritis; TCZ, tocilizumab; MTX, methotrexate

Estimates of the probability of discontinuation due to secondary loss of efficacy or to adverse events in CDAI50 responders from the start of maintenance therapy were computed using the CIF in order to consider the presence of competing risks.^4^, ^5^ Fine‐Gray competing risks regression analysis was used to calculate hazard ratios for TCZ discontinuation associated with MTX use during maintenance therapy. Potential confounders used in Fine‐Gray regression analyses were selected based on the clinical relevance and importance of each variable. Among the potential confounders, I included CDAI >22 (high CDAI) at the start of TCZ treatment (an indicator of initial disease activity) as well as CDAI >10 (high or moderate CDAI) at 6 and 12 months into induction therapy (indicators of residual disease activity at or before the start of maintenance therapy). According to Gray's test, there was no significant impact of MTX use on the cumulative incidence of TCZ discontinuation due to efficacy loss or adverse events. In the Fine‐Gray competing risk regression models, high and moderate residual disease activity at the start of maintenance therapy (namely CDAI >10 at 12 months of induction therapy) and age were predictive factors for TCZ discontinuation due to efficacy loss and adverse events, respectively. I concluded that MTX use is not necessarily relevant to whether the improvements achieved in the first year are maintained during maintenance therapy.

First, if I had evaluated the cumulative incidence of TCZ discontinuation from the start of TCZ treatment for all participants and included CDAI >10 at 6 and 12 months in my Fine‐Gray models as potential confounders, I would have to admit that the authors' criticism of my study was valid. For an analysis including such time‐varying covariates, it may be appropriate to use a time‐dependent regression model in which follow‐up time is divided into different time windows.^6^ In my study, however, as mentioned above, CDAI >10 at 6 and 12 months were determined before I began to follow the CDAI50 responders. CDAI >10 at 6 and 12 months were used as indicators of residual disease activity in these subjects. Thus, these variables were fixed baseline confounders for treatment effect on TCZ discontinuation during maintenance therapy.

Second, I understand that it is inappropriate to adjust for a covariate that is or can be a result of the risk factor under study in analyses of time‐to‐event data.^7^, ^8^ In my study, however, CDAI >10 as measured prior to follow‐up was an effect not of the treatment during maintenance therapy but rather of the treatment during induction therapy prior to follow‐up. In addition, I confirmed that there were no significant differences in rates of CDAI >10 at 6 or 12 months between the monotherapy and combination therapy groups. Statistically speaking, it would have been optimal to randomly assign the CDAI50 responders to either monotherapy or combination therapy group at the start of follow‐up. This was ethically impossible, however, because some patients had chronic kidney disease.

I feel that the conclusions drawn by Austin et al should be interpreted cautiously. Of course, CDAI values themselves change during TCZ treatment. In my study, however, I used CDAI values measured at 0, 6, and 12 months after the start of induction therapy as the baseline characteristics of CDAI50 responders (as indicators of initial disease activity and residual disease activity prior to the start of maintenance therapy); only after these measurements had been recorded did I start follow‐up with these subjects. Generally, CDAI values are among the important baseline characteristics of RA patients; they are expected to become even more commonly used as indicators of residual disease activity at the end of the initial phase of RA treatment in studies addressing predictive factors for drug retention as well as outcomes of drug tapering and/or discontinuation in responders to induction therapy. The authors' assumption that CDAI as determined prior to follow‐up was a time‐varying covariate during follow‐up was mistaken. This error could lead to misunderstanding among readers and confusion among researchers in rheumatology.

## CONFLICT OF INTEREST

The author has declared that no conflicts of interest exist.
